# TkNACs Heterodimerization and Methyl Jasmonate Signaling Synergistically Mediate Root Development in *Taraxacum kok-saghyz*

**DOI:** 10.3390/plants15121923

**Published:** 2026-06-22

**Authors:** Changping Zhang, Yixuan Lin, Ziting Chen, Xiaodong Li, Yuya Geng, Jialong Sun, Lu Qiao, Xifeng Chen, Jie Yan

**Affiliations:** 1 Key Laboratory of Xinjiang Phytomedicine Resource and Utilization of Ministry of Education, College of Life Sciences, Shihezi University, Shihezi 832003, China; 15009357340@163.com (C.Z.); 17590516886@163.com (Y.L.); 17860272271@163.com (Z.C.); xiaodongli202407@163.com (X.L.); 17609930813@163.com (Y.G.); 13031329461@139.com (J.S.); 18993215217@163.com (L.Q.); 2 Xinjiang Production and Construction Corps Key Laboratory of Oasis Town and Mountain-Basin System Ecology, College of Life Sciences, Shihezi University, Shihezi 832003, China

**Keywords:** *Taraxacum kok-saghyz* (*T. kok-saghyz*), NAC transcription factors, methyl jasmonate (MeJA), heterodimerization, root development

## Abstract

*Taraxacum kok-saghyz* (*T. kok-saghyz*) is a promising alternative crop for natural rubber production, in which root development is closely associated with rubber synthesis; however, the molecular mechanisms governing root architecture formation remain largely unclear. NAC transcription factors play pivotal roles in plant root development, yet their functions in *T. kok-saghyz* have not been systematically investigated. In this study, a genome-wide analysis identified 34 NAC family members in *T. kok-saghyz*. Through transcriptomic analysis following methyl jasmonate (MeJA) treatment, 27 genes significantly responsive to MeJA signaling were screened. Sequence analysis revealed that all TkNAC proteins contain a conserved NAM domain. Subcellular localization assays confirmed that TkNAC16, TkNAC20, TkNAC23, and TkNAC30 are localized to the nucleus. Yeast two-hybrid and bimolecular fluorescence complementation assays demonstrated that TkNAC16/18/20/23/30 can form extensive heterodimers. Overexpression lines of *T. kok-saghyz* exhibited significantly increased root length, while leaf growth exhibited line- and stage-specific effects. Collectively, this study provides the first systematic identification of the NAC transcription factor family in *T. kok-saghyz*, elucidates their involvement in methyl jasmonate signaling responses, the construction of heterodimerization networks, and the positive regulation of root elongation. These findings provide crucial genetic resources and a theoretical basis for dissecting the molecular mechanisms underlying the coordinated improvement of root development and rubber yield in *T. kok-saghyz*.

## 1. Introduction

Natural rubber (cis-1,4-polyisoprene), owing to its excellent elasticity, abrasion resistance, and tear strength, has become an indispensable strategic raw material in sectors such as transportation, healthcare, and national defense, with over 50,000 products worldwide relying on its application. However, the current global commercial supply of natural rubber depends almost exclusively on *Hevea brasiliensis* (*H. brasiliensis*) [1]. This single-source production system is facing severe challenges: *H. brasiliensis* cultivation is confined to tropical regions, requires a growth period of 5–8 years before tapping, possesses a narrow genetic background, and is highly vulnerable to fungal pathogens such as South American leaf blight [2]. Furthermore, allergenic proteins present in *H. brasiliensis* latex have caused increasing type I allergic reactions, further exacerbating uncertainty in the industry [3]. Consequently, developing alternative natural rubber-producing plant resources with independent intellectual property rights has become an urgent necessity to secure the global natural rubber supply chain.

*Taraxacum kok-saghyz* (*T. kok-saghyz*), a perennial herbaceous plant in the Asteraceae family, has recently been recognized as one of the most promising alternative crops to *H. brasiliensis* [4]. Compared with *H. brasiliensis*, *T. kok-saghyz* offers outstanding advantages for industrial application: a short growth cycle (harvestable within 6–8 months, enabling planting and harvesting in the same year and dramatically shortening breeding and production cycles); broad adaptability and strong stress tolerance, allowing cultivation in temperate regions and overcoming the geographical constraints of tropical farming [4]; high-quality natural rubber accumulated in its roots; and a well-established genetic transformation system suitable for functional gene studies and molecular breeding [5]. More importantly, a recent study using CRISPR/Cas9 to simultaneously knock out the key inulin biosynthesis genes *Tk1-SST* and *Tk1-FFT* demonstrated that carbon flux reallocation could more than double rubber content in *T. kok-saghyz* roots [6]. This breakthrough clearly illustrates the great potential of molecular breeding strategies to substantially increase rubber yield. Nevertheless, despite the significant application potential of *T. kok-saghyz* in natural rubber production, its industrial progress still faces a core bottleneck: the synergistic regulatory mechanism governing root development and rubber biosynthesis remains unclear. The root system of *T. kok-saghyz* functions not only in nutrient absorption, but also as the primary site for rubber synthesis and storage; thus, root architecture directly determines plant stress tolerance and rubber yield [7]. Studies have shown that root morphological parameters such as root biomass and lateral root density are positively correlated with rubber accumulation [8]. Therefore, elucidating the molecular mechanisms controlling root development in *T. kok-saghyz* and clarifying their synergistic regulatory network with rubber biosynthesis pathways holds both theoretical value and practical significance for the genetic improvement of yield traits in this crop.

In recent years, research directions in *T. kok-saghyz* genomics have gradually evolved from studying a single genome to investigating multiple levels, including genetic variation maps, population evolution, and functional gene identification. In terms of genome assembly, Lin et al. used PacBio and Hi-C technologies to construct the first chromosome-scale reference genome [9]; Luo et al. identified a large number of rubber yield-related SNP markers based on the root transcriptome sequencing of *T. kok-saghyz* [10]. In terms of genetic resources, Yang et al. constructed the first high-density SNP genetic map and mapped six quantitative trait loci (QTL) associated with rubber content [11], and Zhang et al. performed whole-genome sequencing of multiple *T. kok-saghyz* lines to analyze population genetic diversity and reveal the evolutionary pattern of this species [12]. At the same time, genome-wide identification of many transcription factor families (WRKY, GRAS, MADS-box, etc.) has been successfully carried out, confirming the reliability of *T. kok-saghyz* genomic resources in functional gene mining [13,14,15]. In addition, the tissue culture-free CDB genetic transformation system established by Cao et al. provides an efficient tool for subsequent gene function validation [16]. These continuously improving genomic foundations provide a theoretical basis for the genome-wide identification and functional characterization of the NAC transcription factor family in this study.

Jasmonic acid (JA) and its methyl ester derivative methyl jasmonate (MeJA) are core hormonal signaling molecules widely present in plants, playing critical roles in regulating plant growth, development, and stress adaptation [17]. Extensive research has shown that the JA/MeJA signaling pathway not only mediates defense responses to biotic stresses such as mechanical wounding, insect herbivory, and pathogen infection, but also actively participates in adaptive regulation under abiotic stresses including drought, salinity, low temperature, and nutrient deficiency [18,19,20,21,22]. Notably, JA signaling regulates root development in a complex concentration-dependent and tissue-specific manner. Elevated levels of JA and JA-Ile generally inhibit primary root elongation by reducing cell division and expansion in the root meristem via the MYC2-JAZ module [23]. In *T. kok-saghyz*, JA signaling suppresses leaf development, promotes root growth, and enhances natural rubber yield through the COI1-JAZ-MYC2 core module [7]. In *Glycine max*, the transcription factor GmbHLH3 alleviates JA-induced root growth inhibition by suppressing the expression of JA-responsive genes, indicating the existence of a sophisticated negative feedback regulatory mechanism for JA signaling in root growth control [18]. In oat, the JA precursor 12-oxophytodienoic acid (OPDA) modulates root water uptake capacity by inhibiting fine root formation, thereby affecting plant drought tolerance [19]. In *Solanum lycopersicum*, gibberellin counteracts JA signaling to maintain root growth under low-potassium stress [21], revealing a complex crosstalk network between JA and other hormones in shaping root architecture. Furthermore, JA signaling is also involved in regulating root growth under ammonium stress [20] and boron deficiency [22], further confirming its central role as a hub integrating environmental signals and root development. By integrating endogenous and exogenous signals in a concentration- and tissue-specific manner, JA signaling finely regulates root elongation, lateral root initiation, root hair formation, and other aspects of root architecture development, serving as a core regulatory hub linking environmental sensing to root plastic responses.

The root system of *T. kok-saghyz* possesses a unique dual identity: it is not only an important tissue for plant growth, water uptake, and mineral nutrient acquisition but also the primary site for natural rubber synthesis and storage. This duality implies an inherent coupling between root development and rubber biosynthesis. Recent genome-wide transcription factor identification and transcriptomic analysis of root development revealed that thousands of transcription factors exhibit dynamic expression during different stages of root development (10–80 DAP) in *T. kok-saghyz*, with WRKY family genes showing particularly pronounced expression peaks in mature taproots at 72 DAP [24], suggesting that transcriptional regulatory networks play a key role in root maturation and the initiation of rubber biosynthesis. Regarding the regulatory function of JA signaling in rubber biosynthesis, previous studies have provided important clues. In *T. kok-saghyz*, exogenous MeJA treatment significantly induces the expression of core JA pathway components TkCOI1, TkJAZs, and TkMYC2 in latex [7]. Further investigations revealed that TkJAZ proteins physically interact with TkMYC2 in the nucleus, and TkMYC2 activates the transcriptional expression of rubber particle surface proteins encoded by TkSRPP/REF family genes by directly binding to their promoters, thereby promoting natural rubber accumulation [7]. In addition, JA treatment significantly induces the expression of key enzymes involved in rubber biosynthesis, including the core rubber chain elongation enzyme TkCPT [25,26] and the precursor synthase TkGGPS [27,28]. These studies collectively indicate that the JA signaling pathway is a critical upstream regulator of natural rubber biosynthesis in *T. kok-saghyz*. Although the regulatory function of JA signaling in root development has been extensively studied in model plants, how JA signaling specifically regulates root architecture establishment through downstream transcription factor networks in *T. kok-saghyz* remains unclear at the molecular level. In particular, whether there are specific transcriptional regulators for root development and whether JA signaling coordinates root morphogenesis through specific transcription factor modules are scientific gaps that urgently need to be addressed.

NAC (NAM, ATAF1/2, CUC2) transcription factors constitute one of the largest and most functionally diverse transcription factor families in plants, characterized by a highly conserved N-terminal NAC domain of approximately 150 amino acids and a highly variable C-terminal transcriptional regulatory region [29,30,31]. Genome-wide identification studies have revealed significant expansion and diversification of the NAC family across plant species: 115 SxyNAC members classified into 15 subfamilies were identified in the woody plant *Sinojackia xylocarpa*, with promoter regions extensively enriched in light-responsive, hormone-responsive, and stress-responsive cis-elements [29]; 224 NbeNAC members were identified in *Nicotiana benthamiana*, with segmental duplication being the main driver of family expansion [30]; and 85 CtNAC members were identified in *Carthamus tinctorius*, most of which were upregulated under water-deficit conditions, suggesting their involvement in drought responses [31]. These studies demonstrate both the conservation and species-specific expansion of the NAC family during evolution. The functional diversity of NAC transcription factors covers multiple aspects of plant growth and development. In secondary metabolism regulation, *Arabidopsis thaliana AtNAC078* activates the expression of chalcone synthase and other genes under high light intensity, promoting anthocyanin accumulation. In fruit development, *Malus domestica MdNAC1* cooperates with *MdbZIP23* to activate anthocyanin biosynthesis genes [32]. Importantly, NAC transcription factors are key downstream effectors of hormone signaling pathways. In the JA signaling pathway, *Arabidopsis thaliana ANAC019* and *ANAC055* have been identified as JA-inducible transcriptional activators that act downstream of the core transcription factor AtMYC2 to directly regulate the expression of the defense genes *VSP1* and *LOX2* [17]. Further studies demonstrated that the expression of *ANAC019* and *ANAC055* depends on the function of COI1 and AtMYC2, and that the overexpression of *ANAC019* partially rescues the JA-related phenotypes of the atmyc2-2 mutant, confirming the central role of NACs as key downstream regulators of JA signaling [17]. In *Nicotiana tabacum*, *NtNAC028* and *NtNAC080* promote JA accumulation by activating the JA biosynthesis gene *NtLOX3*, thereby regulating leaf senescence [33], indicating a complex bidirectional feedback regulation between NACs and JA signaling.

NAC transcription factors are core regulators of root development in plants, with functions spanning primary root elongation, lateral root initiation, adventitious root formation, and adaptive remodeling of root architecture in response to environmental stresses. In the model plant *Arabidopsis thaliana*, nitrate-induced *NAC056* promotes nitrate assimilation by directly activating the nitrate reductase gene *NIA1*, significantly promoting lateral root growth; nac056 mutants exhibit a marked reduction in lateral root density, whereas overexpression enhances tolerance under low-nitrate conditions [34]. In *Oryza sativa*, the superior haplotype of *OsXND1* (Xylem NAC Domain 1) increases the root surface area, total root length, and convex hull area by 46%, 38%, and 42%, respectively, under drought stress [35], demonstrating the potential of *NAC* genes for improving root drought tolerance. In *Triticum aestivum*, overexpression of *TaRNAC1*, which is highly expressed in roots, significantly increases root length and root biomass while upregulating the gibberellin biosynthesis gene *GA3-ox2*, suggesting that it regulates root growth via the GA pathway [36]. In *Nicotiana tabacum*, topping induces JA signaling, downregulates miR164, and upregulates *NtNAC-R1*, thereby promoting lateral root proliferation and nicotine synthesis [37]. In *Medicago truncatula*, overexpression of *MtNAC969* leads to shorter roots and fewer lateral roots but enhances salt tolerance, whereas RNAi plants exhibit increased lateral root numbers [38]. These cross-species studies consistently indicate that NAC transcription factors are conserved regulators of root architecture establishment. However, in *T. kok-saghyz*, although several gene families such as WRKY [7], CPT [25,26], SRPP/REF [39], GGPS [27], and JAZ/MYC2 [7] have been characterized, genome-wide identification of NAC transcription factors and their functions in root development and rubber biosynthesis remain unexplored. Given that the roots of *T. kok-saghyz* serve the dual functions of nutrient absorption and rubber synthesis/storage, whether NAC transcription factors participate in the coordinated regulation of these two major biological processes is a scientifically important and unanswered question.

The formation of functional modules through homo- or heterodimerization of transcription factors is a critical molecular mechanism for expanding DNA-binding specificity, increasing regulatory target diversity, and fine-tuning downstream gene expression networks. Dimerization of NAC proteins is an important molecular basis for their transcriptional regulatory functions. Of particular interest, heterodimerization between NAC proteins can generate novel functions beyond those of homodimers. In *Nicotiana tabacum*, although NtNAC028 and NtNAC080 share 91.87% amino acid sequence identity, only the heterodimer formed by the two proteins significantly enhances transcriptional activation activity on the target gene *NtLOX3*, whereas homodimers lack this function [33]. This important discovery reveals that NAC heterodimerization acts as a molecular switch for functional innovation, conferring new regulatory properties on NAC transcription factors and expanding their functions in hormone signaling and developmental regulation. In the JA signaling pathway, *AtNAC019* and *AtNAC055* function as transcriptional activators downstream of *AtMYC2* to coordinately regulate defense gene expression [17], but whether they function through heterodimerization requires further validation.

Although the above studies have demonstrated the critical roles of NAC transcription factors in JA signaling and root development, and heterodimerization is an important molecular basis for expanding NAC protein functions, in *T. kok-saghyz*—a crop that integrates root development and natural rubber synthesis—whether NAC family members respond to JA signaling, whether they form transcriptional regulatory modules through homo- or heterodimerization, and whether such modules coordinately participate in root architecture establishment and rubber metabolism regulation have not yet been reported. Critically, genome-wide identification of the NAC family in *T. kok-saghyz*, screening of JA-responsive members, dissection of protein–protein interaction networks, and validation of their biological functions all represent uncharted territory. Therefore, systematic characterization of the NAC transcription factor family in *T. kok-saghyz*, clarification of their responsiveness to JA signaling, and elucidation of heterodimerization patterns among family members are prerequisites for deeply understanding how JA signaling integrates the two major biological processes of root development and rubber biosynthesis.

Based on the above scientific questions, this study first performed genome-wide identification and phylogenetic analysis of the NAC transcription factor family in *T. kok-saghyz* and screened candidate members responsive to MeJA treatment. Subsequently, using yeast two-hybrid and bimolecular fluorescence complementation assays, we demonstrated extensive heterodimerization interactions among TkNAC16, TkNAC20, TkNAC23, and TkNAC30 proteins both in vitro and in living plant cells. On this basis, we generated and obtained transgenic *T. kok-saghyz* lines overexpressing each of these four genes and found that their overexpression significantly promotes primary root elongation while leaf growth exhibits line- and stage-specific effects, indicating that these NAC members act as positive regulators of root development in *T. kok-saghyz.* This study aims to elucidate the molecular mechanism by which NAC transcription factors downstream of JA signaling cooperatively regulate root development in *T. kok-saghyz* via heterodimerization modules, providing a theoretical basis and genetic resources for understanding the function of JA-NAC modules in the coordinated improvement of root architecture and rubber yield.

## 2. Results

### 2.1. Identification of the NAC Gene Family in Taraxacum kok-saghyz and Screening of MeJA-Responsive Members

To systematically identify the NAC transcription factor family in *Taraxacum kok-saghyz* (*T. kok-saghyz*) and mine candidate genes responding to methyl jasmonate (MeJA) signaling, genome-wide data of *T. kok-saghyz* from the National Bioinformatics Center database were used. A combined strategy was employed: a hidden Markov model (HMM) search (PF02365) against the whole genome, together with homology-based retrieval using the annotated NAC protein sequences of *Arabidopsis thaliana* as queries via TBtools. The obtained candidate sequences were merged and duplicates removed, followed by domain validation using the SMART online tool. Sequences containing the complete conserved NAC domain were retained, resulting in the identification of 34 TkNAC family members.

Based on the MeJA-treated transcriptomic data previously generated in our laboratory, differential expression analysis of the 34 *TkNAC* genes was performed using the Majorbio Cloud Platform. In combination with correlation analysis of target genes involved in natural rubber biosynthesis, 27 *TkNAC* genes showing significant expression differences were further selected as candidate genes (i.e., the members that showed significant expression responses to MeJA treatment). The remaining seven *TkNAC* genes (*TkNAC10*, *TkNAC12*, *TkNAC17*, *TkNAC18*, *TkNAC21*, *TkNAC26*, and *TkNAC34*) did not exhibit significant changes in expression levels under MeJA treatment (with |log_2_(fold change)| ≤ 1 and pval ≥ 0.05). It is speculated that these members may not be involved in the response to the MeJA signaling pathway, and therefore they were not included in subsequent in-depth functional analyses. To characterize the basic physicochemical properties of the TkNAC family proteins, systematic predictions were conducted for amino acid length, molecular weight, isoelectric point (pI), grand average of hydropathicity (GRAVY), and subcellular localization of the 27 TkNAC proteins (Table 1). The results showed that the amino acid lengths of TkNAC proteins ranged from 232 to 648 aa, with molecular weights ranging from 26,827.98 to 73,599.35 Da. The pI values ranged from 4.45 to 8.82, among which most members had pI < 7, indicating acidic proteins, while a few were basic proteins. All TkNAC proteins exhibited negative GRAVY values, indicating that all members of this family are hydrophilic proteins, consistent with the typical physicochemical properties of transcription factors. Subcellular localization predictions revealed that the vast majority of TkNAC proteins were localized to the nucleus, consistent with their function as transcription factors regulating downstream gene expression; a few members were predicted to localize to the cytoplasm, chloroplasts, peroxisomes, or vacuoles, suggesting that some TkNAC proteins may have additional biological functions beyond transcriptional regulation.

**Figure 1:**
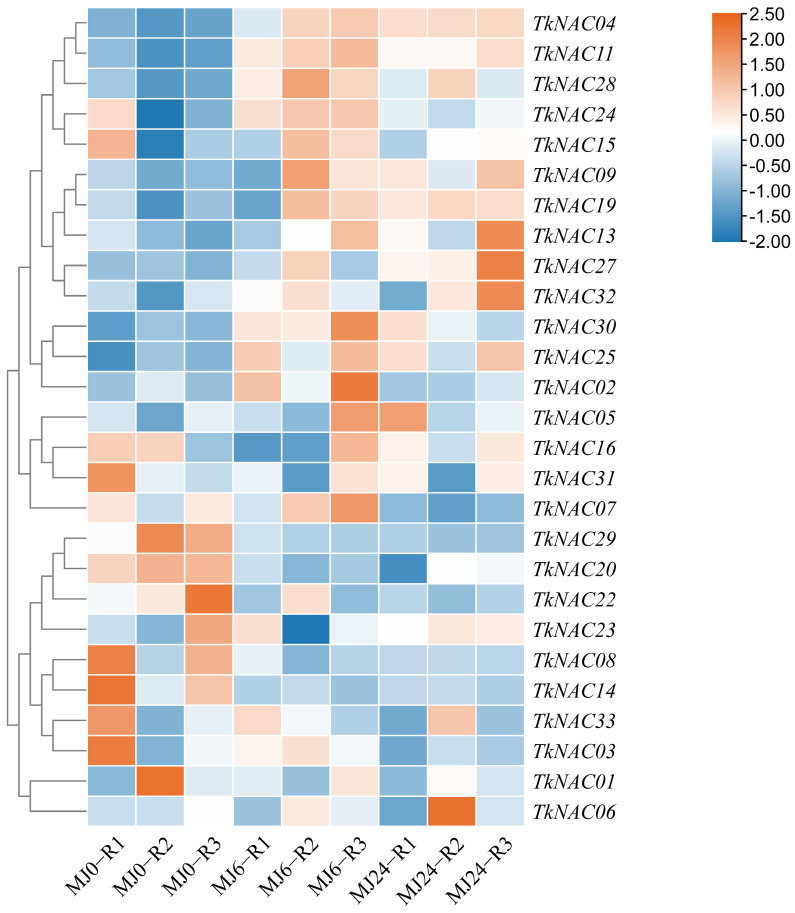
Expression heatmap of *TkNAC* genes in *T. kok-saghyz* roots under MeJA treatment. The heatmap was generated based on transcriptomic data of *T. kok-saghyz* roots at 0 h (MJ0), 6 h (MJ6), and 24 h (MJ24) after MeJA treatment. The color scale represents log_2_-transformed relative expression levels, with red indicating high expression and blue indicating low expression.

To investigate the response characteristics of TkNAC family members to MeJA signaling, the expression levels of the 27 candidate genes in roots at different time points (0, 6, and 24 h) after MeJA treatment were analyzed using the aforementioned transcriptomic data, and an expression heatmap was generated (Figure 1). Cluster analysis revealed that the *TkNAC* genes exhibited time-dependent differential expression patterns in response to MeJA treatment, which could be classified into several expression clusters. Some genes (e.g., *TkNAC04*, *TkNAC11*, *TkNAC09*, *TkNAC19*) were upregulated after MeJA treatment, with expression levels peaking at 16 and 24 h post-treatment; another group of genes (e.g., *TkNAC08*, *TkNAC14*, *TkNAC33*, *TkNAC03*) showed an overall downregulation trend after treatment and were mainly highly expressed at 0 h. In addition, some genes exhibited transient response characteristics, for example, *TkNAC24* was specifically highly expressed at 6 h post-treatment, while *TkNAC13*, *TkNAC27*, and *TkNAC32* were induced to high expression at 24 h post-treatment. These results indicate that different TkNAC members display distinct temporal dynamics in their response to MeJA signaling.

### 2.2. Phylogenetic Analysis of the TkNAC Gene Family

**Figure 2:**
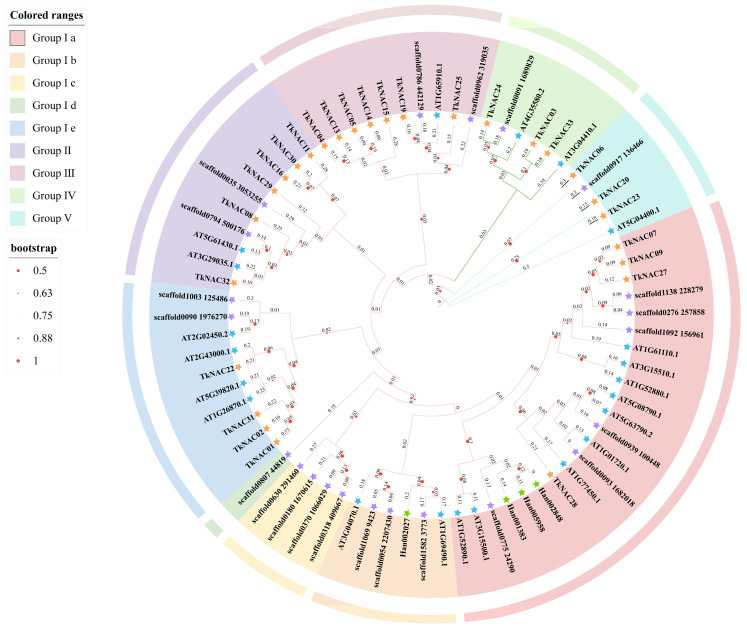
Phylogenetic tree of TkNAC proteins and NAC proteins from related species. The phylogenetic tree was constructed using the neighbor-joining (NJ) method, and bootstrap values are indicated at the nodes. Orange stars represent *T. kok-saghyz*, blue stars represent *Arabidopsis thaliana*, purple stars represent *Hevea brasiliensis*, and green stars represent *Helianthus annuus*.

To elucidate the evolutionary relationships and functional divergence of the NAC gene family in *T. kok-saghyz*, full-length NAC protein sequences from *T. kok-saghyz*, *Arabidopsis thaliana*, *Hevea brasiliensis* (*H. brasiliensis*), and *Helianthus annuus* were selected, and a phylogenetic tree was constructed using the neighbor-joining (NJ) method (Figure 2). The results showed that all NAC proteins could be divided into six subfamilies (Groups I–VI). The NAC family members of *T. kok-saghyz* were unevenly distributed among the subfamilies. Among them, Group I a contained the largest number of members, with proteins such as TkNAC07, TkNAC09, and TkNAC27 clustered in this group. Notably, TkNAC16 and TkNAC30 clustered closely together, forming a distinct small branch, indicating a very close phylogenetic relationship and suggesting that this branch is unique to *T. kok-saghyz*. This result implies that this branch may have undergone species-specific expansion during evolution, and its members are speculated to participate in biological processes unique to rubber dandelion.

From the perspective of interspecific evolutionary relationships, NAC members of *T. kok-saghyz* exhibited close clustering with those of *Helianthus annuus*, which belongs to the same Asteraceae family. For example, TkNAC28 clustered with sunflower NAC proteins in the same branch, reflecting the high evolutionary conservation within Asteraceae species. NAC members of *H. brasiliensis* were mostly concentrated in Group I a and clustered with some *T. kok-saghyz* NAC members in the same branch, suggesting potential functional conservation in the regulatory pathways of rubber biosynthesis between the two species. In addition, a large number of *T. kok-saghyz NAC* genes clustered with homologous sequences from *Arabidopsis*, indicating high conservation in biological functions such as stress responses and growth/developmental regulation. In summary, the NAC family of *T. kok-saghyz* exhibits clear lineage-specific expansion and functional divergence, providing an important evolutionary basis for the further mining of candidate *NAC* genes involved in rubber biosynthesis and stress tolerance regulation in this species.

### 2.3. Analysis of Gene Structure and Promoter Cis-Regulatory Elements of the TkNAC Gene Family

**Figure 3:**
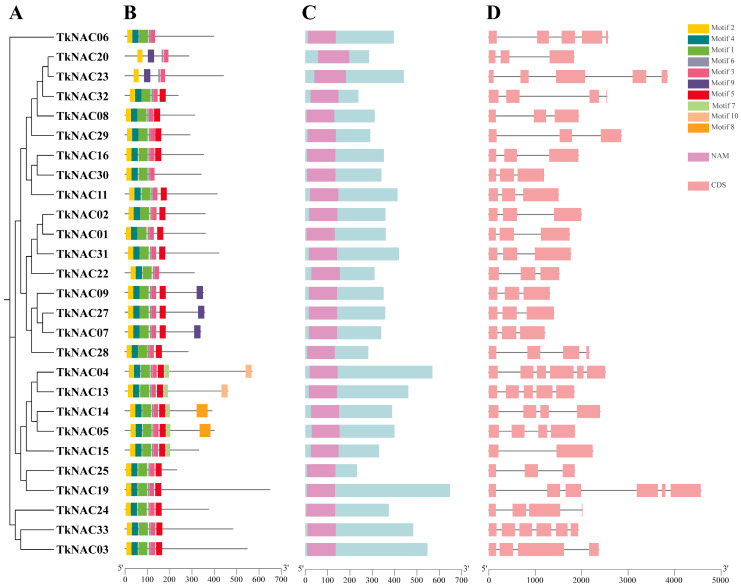
Gene structure and conserved motif analysis of *TkNACs*. (**A**) Phylogenetic tree of the 27 *TkNAC* genes. (**B**) Domain distribution. (**C**) Conserved motif distribution. (**D**) Gene structure.

To investigate the evolutionary conservation and functional divergence of TkNAC transcription factors, systematic analyses of gene structure and promoter cis-acting elements were performed for the 27 *TkNAC* genes. Phylogenetic analysis classified the TkNAC proteins into several distinct evolutionary clades, within which members shared highly similar conserved motif compositions (Figure 3A). Motifs 1–4, which constitute the canonical NAM domain of the NAC family, were highly conserved across almost all TkNAC proteins, indicating their indispensable role in maintaining the core DNA-binding function. Notably, motifs 5–10 were specifically distributed only in certain TkNAC members (e.g., *TkNAC04*, *TkNAC13*, *TkNAC14*, *TkNAC05*), suggesting that these specific motifs drive neofunctionalization during evolution (Figure 3B). Domain analysis showed that all *TkNAC* genes contain a conserved NAM domain, which is predominantly located at the 5′ end of the genes, with variations in domain length among different members (Figure 3C).

Gene structure analysis revealed significant divergence in the exon–intron organization patterns among TkNAC family members: intron lengths varied, and the exon/intron arrangement was highly conserved within the same evolutionary clade but markedly diverged among different clades, suggesting that gene structure variation may be associated with functional diversification of the family (Figure 3D). Furthermore, genes with close phylogenetic relationships exhibited highly similar gene structures, further validating the reliability of the evolutionary classification in this study.

**Figure 4:**
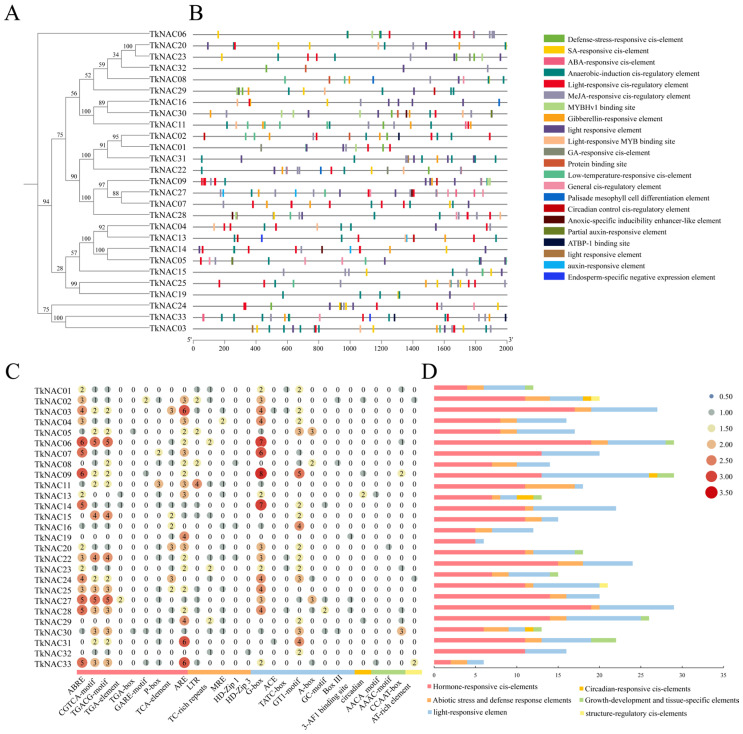
Promoter cis-acting elements of *TkNACs*. (**A**) Phylogenetic tree of the 27 TkNACs. (**B**) Distribution of cis-acting elements in the promoters. (**C**) Statistics of the number of each cis-element. (**D**) Classification statistics of cis-elements.

To dissect the potential regulatory mechanisms of *TkNAC* genes, cis-acting elements in the 2000 bp promoter regions upstream of the 27 *TkNACs* were identified (Figure 4). The results showed that the *TkNAC* promoters were enriched with numerous cis-elements involved in hormone responses, stress defense, light signaling, and developmental regulation. Among these, abscisic acid (ABA) and MeJA response elements were the most abundant, suggesting that *TkNAC* genes primarily regulate stress responses through the ABA and MeJA signaling pathways. The widespread presence of salicylic acid (SA), gibberellin (GA), and auxin-related elements also indicated their involvement in growth and developmental processes. In addition, various stress-responsive elements, including those for low temperature, anaerobic conditions, and defense stress, as well as ubiquitously present light-responsive elements, were identified in the promoters, indicating that this family plays important roles in abiotic/biotic stress responses and light signaling regulation. Simultaneously, elements associated with cell differentiation, circadian rhythms, and tissue-specific expression were detected, confirming the pleiotropic functions of these genes. Notably, the types and numbers of cis-elements varied significantly among different TkNAC members, representing the molecular basis for the functional divergence of paralogous genes. Among them, *TkNAC07* and *TkNAC33* exhibited the highest abundance of ABA/MeJA elements; *TkNAC06*, *TkNAC09*, *TkNAC14*, and *TkNAC28* were enriched with numerous stress-related elements; *TkNAC07*, *TkNAC09*, and *TkNAC31* showed significant enrichment of light-responsive elements; and *TkNAC06*, *TkNAC14*, and *TkNAC33* were rich in development-related elements, indicating differentiation in the regulatory landscape of the TkNAC family.

### 2.4. Analysis of Tissue-Specific Expression Patterns and Subcellular Localization Characteristics

**Figure 5:**
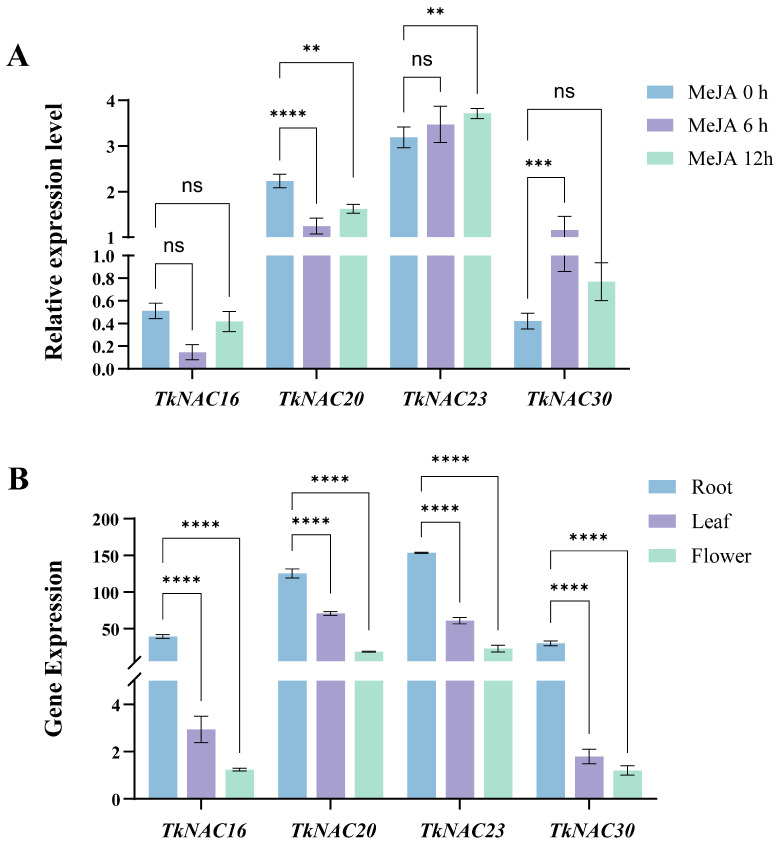
Expression patterns of *TkNAC16/20/23/30*. (**A**) The relative expression levels of the four *TkNAC* genes under MeJA treatment. (**B**) Tissue-specific expression analysis of the four *TkNAC* genes. ns indicates not significant; ** *p* < 0.01; *** *p* < 0.001; **** *p* < 0.0001.

To preliminarily dissect the biological functions of TkNAC transcription factors, four genes—*TkNAC16*, *TkNAC20*, *TkNAC23*, and *TkNAC30*—were ultimately selected for functional analysis based on the above screenings. Their expression levels were examined at 0, 6, and 24 h after jasmonic acid treatment (Figure 5A). The results showed that all four genes exhibited temporal responses to JA, indicating their involvement in JA-mediated signaling pathways. Among them, *TkNAC16* showed relatively high basal expression at 0 h, which decreased at 6 h; *TkNAC20* was highly expressed at 0 h and continuously declined at 6 h and 24 h, displaying a negative regulation pattern; *TkNAC23* had extremely low expression at 0 h; *TkNAC30* showed low expression at 0 h but high expression at 6 h. Tissue expression analysis (Figure 5B) revealed that all four genes exhibited the highest expression in roots, followed by leaves, and the lowest in flowers (*p* < 0.0001), indicating that the root is their primary functional tissue. Among them, *TkNAC23* showed the strongest root specificity; *TkNAC20* had significantly higher expression in leaves compared to *TkNAC16/23/30*; *TkNAC16* and *TkNAC30* displayed similar root-dominant expression patterns.

**Figure 6:**
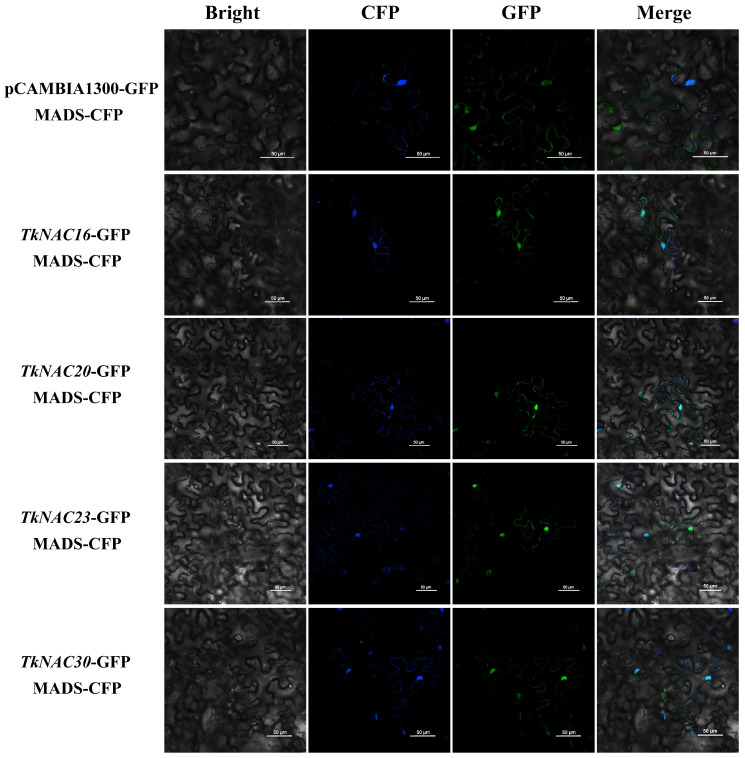
Subcellular localization of TkNAC16/20/23/30 proteins. pCAMBIA1300-GFP served as the empty vector control, and MADS-CFP was used as the nuclear localization marker. Bright: bright field; CFP: cyan fluorescent channel; GFP: green fluorescent channel; Merge: merged fluorescent channels. Scale bar = 100 μm.

Subcellular localization results (Figure 6) showed that the GFP signal of the empty vector control was diffusely distributed throughout the cytoplasm. In contrast, the fusion proteins of TkNAC16, TkNAC20, TkNAC23, and TkNAC30 with GFP exhibited fluorescence signals that largely overlapped with those of the nuclear marker MADS-CFP, appearing as cyan fluorescence, confirming that all four TkNAC proteins are localized to the nucleus and possess the molecular basis for functioning as transcription factors to regulate downstream gene transcription.

### 2.5. Analysis of Protein–Protein Interactions Among TkNAC16, TkNAC20, TkNAC23, and TkNAC30

**Figure 7:**
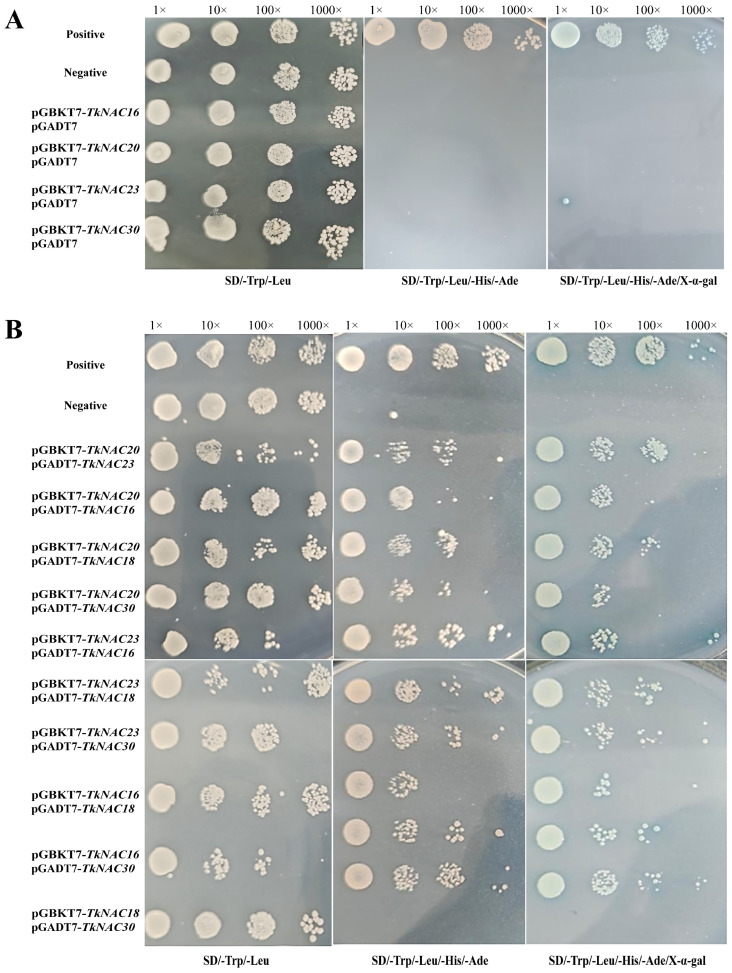
Validation of protein–protein interactions among TkNAC16/20/23/30 by yeast two-hybrid assay. (**A**) Detection of self-activation activity of bait vectors. (**B**) Validation of TkNAC protein interactions. Positive control: pGBKT7-p53 + pGADT7-T; negative control: pGBKT7-lam + pGADT7-T.

To determine whether TkNAC16, TkNAC20, TkNAC23, and TkNAC30 proteins exert their transcriptional regulatory functions through homo-/heterodimerization, systematic analyses of protein–protein interactions were performed using yeast two-hybrid (Y2H) and bimolecular fluorescence complementation (BiFC) assays, both in vitro and in living plant cells. First, bait vectors pGBKT7-*TkNAC16/20/23/30* and prey vectors pGADT7-*TkNAC16/18/20/23/30* were constructed. To test for self-activation activity, each bait vector was co-transformed into yeast competent cells together with the empty pGADT7 vector. The results showed that on quadruple dropout (SD/-Trp/-Leu/-His/-Ade) selection medium, neither the negative control nor any of the TkNAC16/20/23/30 bait–empty combinations could grow, nor did they exhibit X-α-gal coloration, demonstrating that none of the four TkNAC proteins possess autonomous transcriptional activation activity (Figure 7A). Subsequently, Y2H interaction screening was performed by co-transforming different bait–prey combinations into yeast cells, followed by cultivation after gradient dilution. The results showed that combinations such as TkNAC20-TkNAC23/16/18/30, TkNAC23-TkNAC16/18/30, and TkNAC16-TkNAC18/30 grew normally on quadruple dropout medium and exhibited blue coloration, confirming direct interactions among these proteins in vitro. In contrast, the TkNAC18–TkNAC30 combination failed to grow, indicating no direct interaction between this pair of proteins (Figure 7B).

**Figure 8:**
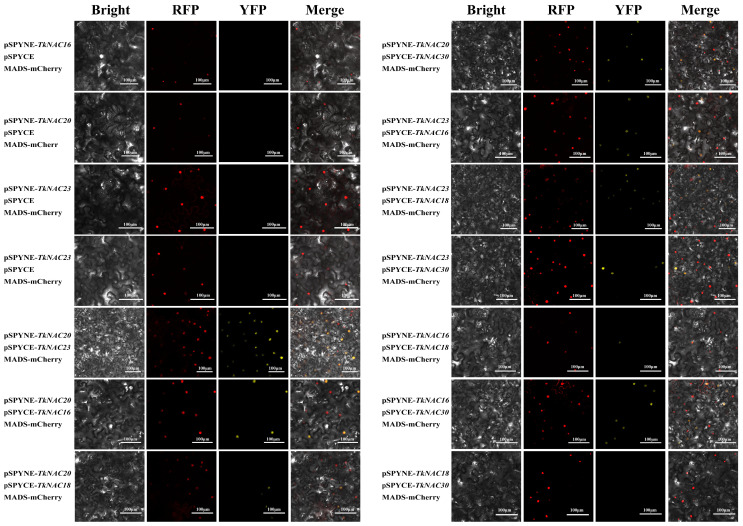
Validation of protein–protein interactions among TkNAC16/20/23/30 by BiFC assay. MADS-mCherry was used as a nuclear localization marker. Bright: bright field; YFP: 514 nm; RFP: 561 nm; Merge: merged fluorescent channels. Scale bar = 100 μm.

To validate the authenticity of these interactions in living plant cells, BiFC expression vectors were constructed. The corresponding *TkNAC* genes were fused to the pSPYNE and pSPYCE vectors, respectively. Plasma membrane-localized marker protein MADS-mCherry was used as a subcellular localization reference, and the constructs were transiently transformed into Nicotiana benthamiana. Confocal laser scanning microscopy observations (Figure 8) revealed that interaction combinations including TkNAC20 with TkNAC23/18/30/16, TkNAC23 with TkNAC16/18/30, and TkNAC16 with TkNAC18/30 produced strong orange-yellow fluorescence signals in the nucleus, whereas the negative control and the TkNAC18–TkNAC30 combination showed no fluorescence signals, which was highly consistent with the Y2H results. Together, these results confirm that TkNAC family members can form heterodimers in the nucleus and cooperatively participate in the transcriptional regulation of downstream genes through protein–protein interaction modules, providing evidence for elucidating the molecular mechanism by which NAC transcription factor family members coordinately regulate growth, development, and stress responses in *T. kok-saghyz*.

### 2.6. Generation and Expression Analysis of TkNAC16/20/23/30 Overexpression Lines

**Figure 9:**
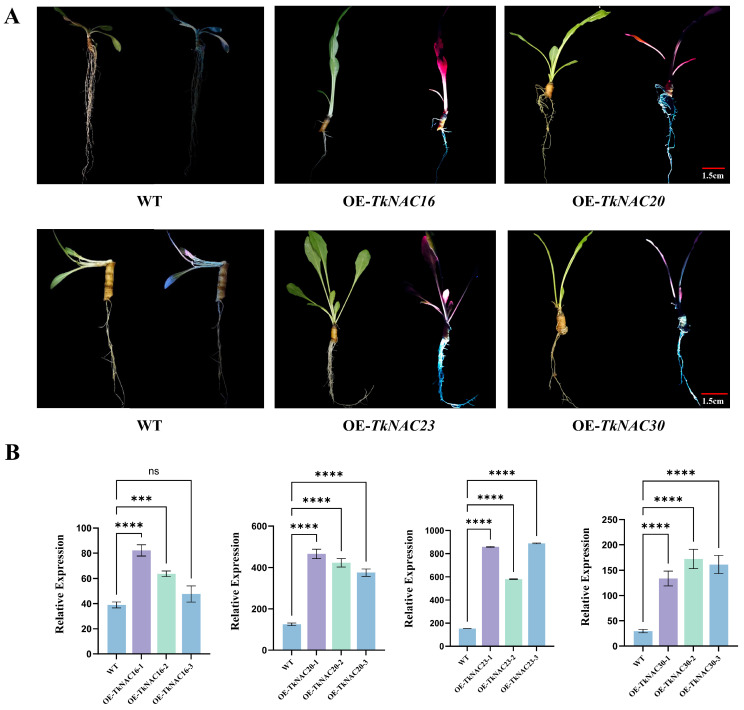
Identification and analysis of *TkNAC16/20/23/30* overexpression lines. (**A**) GFP fluorescence observation of the OE-*TkNAC16/20/23/30* overexpression lines. Bar = 1.5 cm. (**B**) qRT-PCR analysis of root tissues from wild-type and overexpression lines. ns: no significant difference; *** *p* < 0.001; ****p < 0.0001.

To dissect the biological functions of TkNAC family members in the growth, development, and stress responses of *T. kok saghyz*, the coding sequences of *TkNAC16*, *TkNAC20*, *TkNAC23*, and *TkNAC30* were individually cloned into the plant expression vector pCAMBIA-1300-eGFP. Transgenic overexpression lines (OE-*TkNAC16*, OE-*TkNAC20*, OE-*TkNAC23*, and OE-*TkNAC30*) were generated via *Agrobacterium rhizogenes*-mediated root-cutting infiltration method. Positive identification of regenerated plants was performed by GFP fluorescence imaging. The results showed that compared with the wild-type control, each overexpression line exhibited clear green fluorescence signals, confirming that the exogenous *TkNAC* genes had been successfully integrated into the genome of *T. kok-saghyz* and were stably expressed (Figure 9A). Subsequently, the relative expression levels of the target genes in each transgenic line were examined by quantitative real-time PCR. The results showed that the transcript levels of the corresponding *TkNAC* genes in all overexpression lines were extremely significantly upregulated compared with the wild-type control (*p* < 0.0001). Among them, the OE-*TkNAC23* line exhibited the greatest increase in gene expression, followed by the OE-*TkNAC16*, OE-*TkNAC20*, and OE-*TkNAC30* lines; significant differences in expression levels were observed among some independent lines, whereas no significant differences were detected among wild-type lines (Figure 9B). In this study, *T. kok-saghyz* overexpression lines for the four *TkNAC* genes were successfully obtained, and the target genes were efficiently overexpressed in the transgenic plants, providing reliable genetic material for subsequent functional validation, phenotypic characterization, and the mechanistic dissection of *TkNAC16/20/23/30*.

### 2.7. Overexpression of TkNAC16/20/23/30 Significantly Alters Root Phenotype in T. kok-saghyz

**Figure 10:**
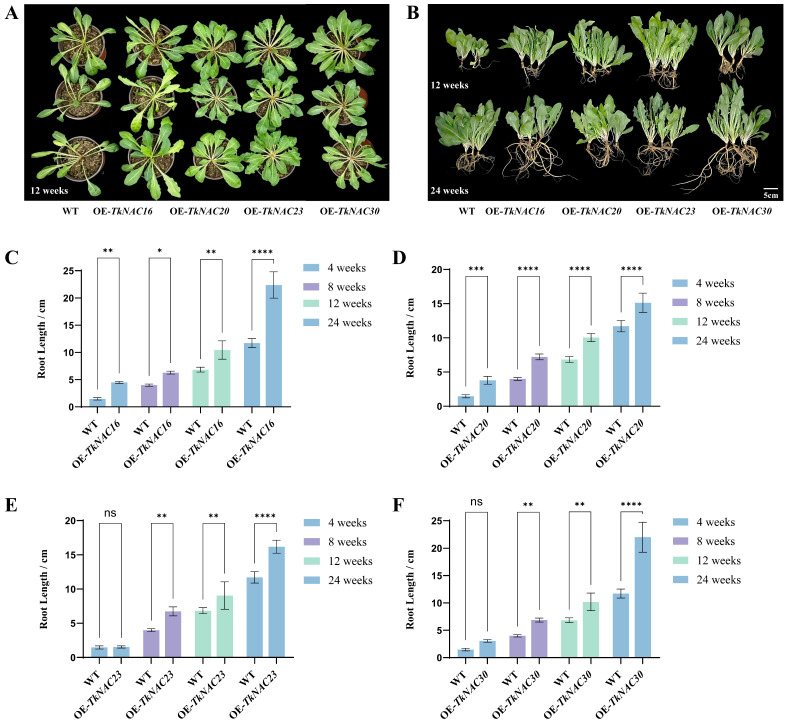
Overexpression of *TkNAC16/20/23/30* significantly promotes root growth in *T. kok-saghyz*. (**A**,**B**) Phenotypic observation of overexpressing plants at different growth stages. Scale bar = 5 cm. (**C**–**F**) Measurement of root length in overexpression lines at different growth stages. ns: no significant difference; * *p* < 0.05; ** *p* < 0.01; *** *p* < 0.001; **** *p* < 0.0001.

To elucidate the biological functions of *TkNAC16*, *TkNAC20*, *TkNAC23*, and *TkNAC30* in the root development of *T. kok-saghyz*, transgenic lines were generated via overexpression vector construction, followed by systematic phenotypic observation and quantitative root length analysis at 4, 8, 12, and 24 weeks after planting. The leaf growth of overexpression lines exhibited line- and stage-specific effects (Figure S1 and Figure 10A). Root development in all overexpression lines was significantly better than that in WT, and the difference in root length between overexpression lines and WT gradually increased with growth time (Figure 10B). Quantitative measurement of root length further confirmed (Figure 10C–F) that root elongation was significantly promoted in all four overexpression lines throughout the 24-week growth period. At week 4, OE-*TkNAC23/30* showed no significant difference from the wild type, but starting from week 8, root length in all overexpression lines became significantly longer than that of the wild type, and the difference continued to widen as growth progressed. Notably, at week 24, OE-*TkNAC16/30* exhibited the strongest promotion of root elongation, followed by OE-*TkNAC20/23*. Collectively, these results demonstrate that *TkNAC16/20/23/30* act as positive regulators of root elongation in *T. kok-saghyz*, and their overexpression promotes root development, providing a genetic foundation for further dissecting the molecular mechanisms underlying root architecture establishment in this species.

## 3. Discussion

NAC transcription factors are among the largest and most functionally diverse families in plants, playing critical roles in growth regulation and stress responses [40,41,42]. Although the NAC family has been characterized in various plant species, its systematic identification and functional analysis in *Taraxacum kok-saghyz* (*T. kok-saghyz*), a highly promising alternative natural rubber crop, remain unclear. This study fills this gap by revealing, for the first time, the molecular mechanism by which TkNAC16, TkNAC20, TkNAC23, and TkNAC30, as key downstream regulators of jasmonic acid (JA) signaling, synergistically promote root elongation through heterodimerization modules. The NAC members of *T. kok-saghyz* exhibited an uneven distribution on the phylogenetic tree. Similar to the distribution pattern of *Hevea brasiliensis* (*H. brasiliensis*) HbNACs, Group I a also represented the largest subfamily in the *T. kok-saghyz* NAC family, and this subfamily may play a specialized role in regulating rubber biosynthesis in rubber-producing plants [43]. In contrast to Yang et al., who focused on the response of HbNACs to cold stress, the present study further uncovers the responsiveness of members of this subfamily to JA signaling, thereby extending the function of Group I a to hormone-mediated developmental regulation. Notably, TkNAC16 and TkNAC30 clustered closely together on the phylogenetic tree, forming a distinct small branch. No orthologous genes with this evolutionary feature have been found in Asteraceae species to date. In comparison, although the sugarcane NAC family also exhibits branch-specific conservation of gene structure [44], all its members have orthologs in monocots. The emergence of this unique branch in *T. kok-saghyz* suggests that TkNAC16/30 may have undergone species-specific neofunctionalization, participating in biological processes unique to rubber dandelion, such as the coordinated regulation of root development and rubber biosynthesis.

Conserved motif analysis indicates that motifs 1–4, which constitute the NAM domain, are highly conserved across all TkNACs, consistent with findings in fruit ripening-related NACs [45] and reflecting the evolutionary stability of the core DNA-binding function of the NAC family. However, motifs 5–10 are specifically distributed only in some members, and this branch-specific distribution may drive functional divergence among TkNAC members. Furthermore, the functions of the NAC family in plant development have been extended to the regulation of seed development and germination [46], while the present study reveals a novel role for NACs in root development, enriching the functional repertoire of this family.

The promoter regions of *TkNACs* are enriched with numerous hormone-responsive cis-elements, among which MeJA and ABA elements are the most abundant, which is highly consistent with the well-established involvement of NACs in hormone signaling pathways [40,47]. Through systematic analysis of promoter cis-element composition, this study clearly demonstrates the widespread distribution of MeJA-responsive elements in *TkNAC* promoters, indicating that JA signaling is a core upstream pathway regulating *TkNAC* expression. This finding aligns with studies on the mechanism by which MeJA induces the switch from growth to defense in plants [48], as well as with observations that MeJA enhances cold tolerance by inducing NAC gene expression [49] and activates ethylene synthesis [50]. Moreover, in *Vigna angularis*, NAC genes have been demonstrated to participate in rust resistance through the JA signaling pathway [51], further supporting the universality of the JA-NAC regulatory module in plant defense and development.

Tissue-specific expression pattern analysis revealed that *TkNAC16*/*20*/*23*/*30* all exhibited the highest expression levels in roots. This result is highly consistent with the transcriptomic analysis of transcription factor expression profiles during root development in *T. kok-saghyz* by Su et al., although that study did not functionally validate the NAC transcription factors [24]. The present study, building on this foundation, further confirms the biological functions of these four genes. Subcellular localization assays confirmed that all four proteins are localized to the nucleus, providing a cytological basis for their function as transcription factors. Bimolecular fluorescence complementation and yeast two-hybrid assays revealed a complex heterodimerization network among TkNAC16, TkNAC18, TkNAC20, TkNAC23, and TkNAC30, whereas TkNAC18 and TkNAC30 did not interact. This selective interaction pattern is similar to findings in *Nicotiana tabacum*, where Lu et al. demonstrated that only the NtNAC028/080 heterodimer significantly activates target gene expression, highlighting the specificity of heterodimerization [33]. As proposed by Jensen and Skriver, NAC heterodimerization can finely tune downstream gene networks by altering DNA-binding properties or regulatory activity [52]. However, whether dimer formation indeed changes DNA-binding specificity and transcriptional activation activity needs to be determined through the dual-luciferase assay and EMSA by comparing the transcriptional activation activities of homodimeric and heterodimeric combinations and their binding affinities to typical NAC target sequences. Notably, all four interacting *TkNACs* are MeJA-responsive genes and are highly expressed in roots, which is highly reminiscent of the functional characteristics of *Arabidopsis ANAC019/055* as JA-inducible transcriptional activators acting downstream of *AtMYC2* [17]. This further suggests that in *T. kok-saghyz*, JA signaling activates the expression of specific *TkNACs* as downstream genes of *MYC2*, and these NAC proteins synergistically regulate root development target genes through heterodimerization, thereby mediating the regulation of root architecture by JA signaling.

Analysis of transgenic overexpression lines showed that primary root length in lines overexpressing each of the four *TkNACs* gradually increased during the 8- to 24-week observation period, indicating that these genes specifically promote root development. This root-specific effect is consistent with the functions of several previously reported *NAC* genes, although differences exist in the specific modes of action. For example, overexpression of *OsNAC10* in *Oryza sativa* increases root biomass and enhances grain yield under field drought conditions [53]; overexpression of *GmNAC19* in *Glycine max* promotes both root growth and seed yield [54]; and *Arabidopsis NAC056* promotes lateral root growth [34]. In contrast, *TkNACs* primarily promote primary root elongation, suggesting functional divergence among different NAC members in the regulation of root architecture. Studies on the genetic basis of hairy root formation in *Nicotiana tabacum* have also revealed a role for *NACs* in adventitious root initiation [55]. Similar to the present study, these findings indicate that *NAC* genes are core regulators of root architecture establishment, although the specific root types they regulate exhibit species- and gene-specific differences. Similar to the mechanism by which *Arabidopsis NAC1* maintains root meristem activity [56], TkNAC16/20/23/30 may promote primary root elongation by enhancing cell division in the root apical meristem, but the precise mechanism requires further validation.

The root system of *T. kok-saghyz* serves the dual functions of nutrient absorption and rubber biosynthesis. Previous studies have shown that JA signaling regulates *TkSRPP*/*REF* expression and thus controls rubber synthesis through the TkJAZs-TkMYC2 module [7], while MeJA treatment induces the coordinated expression of rubber biosynthesis genes and root development-related genes [57]. The four *TkNAC* genes identified in this study are both JA-responsive genes and positive regulators of primary root elongation, providing key transcriptional regulatory nodes connecting JA signaling, root development, and rubber biosynthesis. This inference is supported by previous findings showing a co-expression network association between the NAC family and rubber biosynthesis-related genes [58], and the present study advances this association from correlative analysis to functional validation. In addition, the expression patterns of *HbNACs* in response to cold stress in *H. brasiliensis* [43] are functionally complementary to the JA-responsive NACs reported here, together supporting the idea that NACs in rubber-producing plants may possess multiple functions encompassing both stress responses and developmental regulation.

Although this study systematically reveals the functions of TkNAC16/20/23/30 in root development and their heterodimerization regulatory mechanisms, several limitations remain. First, the potential interactions between NACs and core JA signaling components such as JAZs and MYC2 have not yet been explored. Future studies should identify the interactions between TkNACs and JA pathway proteins to fully elucidate the JA-NAC regulatory module. Second, phenotypic validation using loss-of-function mutants is lacking. The use of the CRISPR/Cas9 genome editing system established in *T. kok-saghyz* [59] to generate tknac knockout mutants is warranted. Third, the direct target genes have not yet been identified. Future work should combine ChIP-Seq or DAP-Seq to identify the direct downstream target genes of *TkNACs* at the genome-wide level and construct the transcriptional network regulating root development. Finally, this study did not directly analyze rubber content or rubber particle morphology in the transgenic lines. Future systematic measurements of rubber content, molecular weight distribution, and rubber particle ultrastructure in OE-*TkNACs* lines are needed, with particular attention to whether NACs differentially regulate rubber particle morphology (e.g., size distribution and density). This would hold significant theoretical value and application prospects for understanding the coordinated regulation of rubber yield and quality, and would lay the foundation for a comprehensive elucidation of the molecular mechanism by which the JA-NAC module governs root development and rubber biosynthesis in *T. kok-saghyz*.

## 4. Materials and Methods

### 4.1. Plant Materials

*Taraxacum kok-saghyz* (*T. kok-saghyz*) seeds were collected from the Tekes River Valley in the Ili Prefecture, Xinjiang. The seeds were sterilized with 75% ethanol for 30 s and 2% sodium hypochlorite for 10 min, rinsed five times with sterile water, and then sown on 1/2 MS medium (containing 15 g·L^−1^ sucrose and 7 g·L^−1^ agar, pH 5.8). Germination was carried out under conditions of 21 °C and a 16 h light/8 h dark photoperiod. The seedlings were transplanted into a mixed substrate of peat soil:vermiculite = 2:1 for further cultivation. *Nicotiana benthamiana* seeds were sterilized using the same procedure and sown in the substrate, where they were grown for 3–4 weeks for subcellular localization and bimolecular fluorescence complementation assays. Methyl jasmonate (MeJA) treatment: Six-month-old *T. kok-saghyz* seedlings were sprayed with 0.8 mmol·L^−1^ MeJA, while the control group was sprayed with an equal volume of distilled water. Roots were collected at 0, 6, and 24 h after treatment, immediately frozen in liquid nitrogen, and stored at −80 °C, with three biological replicates per time point.

The bacterial strains included: Escherichia coli DH5α competent cells, *Agrobacterium tumefaciens* GV3101, and K599, all purchased from Hengchao Biotechnology Co., Ltd. (Xinjiang, China). Main reagents: restriction endonuclease Sma*I*, high-fidelity PCR amplification reagent, and EasyScript^®^ One-Step gDNA Removal were purchased from Beijing TransGen Biotech Co., Ltd. (Beijing, China); Plant Total RNA Extraction Kit, Reverse Transcription Kit, Plasmid Mini-Preparation Kit, and DNA Gel Extraction Kit were purchased from Nanjing Vazyme Biotech Co., Ltd. (Nanjing, China); conventional chemical reagents were all domestic analytical grade (China); primers were synthesized and purified by Sangon Biotech (Shanghai) Co., Ltd. (Shanghai, China).

### 4.2. Genome-Wide Identification of the NAC Gene Family in T. kok-saghyz

The whole-genome data and annotation files of *T. kok-saghyz* were downloaded from the National Bioinformatics Center database (https://ngdc.cncb.ac.cn/gwh/; accessed on 15 July 2025). NAC family members were identified using the following two methods: First, the hidden Markov model (HMM) method was employed based on the Pfam accession number (PF02365) of the NAC conserved domain. The Simple HMM Search function of TBtools V2.056 software was used to search for candidate sequences containing the NAC domain in the *T. kok-saghyz* proteome. Second, the protein sequences of *Arabidopsis thaliana* NAC family members were downloaded from the TAIR database (https://www.arabidopsis.org/; accessed on 15 August 2025), and local BLAST alignment against the *T. kok-saghyz* genome was performed using TBtools software. The results obtained from the two methods were merged and redundancy was removed. The SMART online tool (https://smart.embl.de/; accessed on 7 October 2025) was then used to further verify whether the candidate proteins contained a complete NAC conserved domain. Finally, a total of 34 TkNAC family members were identified. The ExPASy ProtParam tool (https://web.expasy.org/protparam/; accessed on 8 October 2025) was used to analyze the amino acid length, molecular weight, isoelectric point, and grand average of hydropathicity (GRAVY) of each protein.

### 4.3. Screening of MeJA-Responsive TkNAC Genes and Phylogenetic Tree Construction

Based on the transcriptome data of *T. kok-saghyz* at different time points (0, 6, 24 h) after MeJA treatment, differential expression screening of the *NAC* gene family was performed using the Majorbio Cloud online transcriptome analysis website (https://cloud.majorbio.com/; accessed on 8 October 2025). The screening criterion was *p*-value ≤ 0.05, and finally, 27 MeJA-responsive *TkNAC* genes were obtained.

To investigate the phylogenetic relationship of NAC proteins in *T. kok-saghyz* and compare their evolutionary relationships with NAC proteins from *Arabidopsis thaliana*, *Hevea brasiliensis*, and *Helianthus annuus*, multiple sequence alignment of NAC protein sequences from *T. kok-saghyz* and the above species was first performed using MEGA 7.0 software. Subsequently, a phylogenetic tree was constructed using the neighbor-joining method with 1000 bootstrap replicates. According to the classification standard of the *A. thaliana* NAC family, the TkNAC members were divided into six subfamilies (Groups I–VI). The phylogenetic tree was visualized using the online website iTOL (https://itol.embl.de/; accessed on 15 October 2025).

### 4.4. Analysis of Gene Structure, Conserved Motifs, and Promoter Cis-Elements of TkNACs

Using the 27 obtained MeJA-responsive NAC protein sequences from *T. kok-saghyz*, a phylogenetic tree of NAC proteins was constructed in TBtools software to obtain the phylogenetic tree file. Subsequently, conserved domain analysis was performed using the Batch-CD-Search tool (https://www.ncbi.nlm.nih.gov/Structure/cdd/wrpsb.cgi/; accessed on 21 October 2025) to obtain the domain information file. Conserved motif analysis was conducted using the MEME online program (https://meme-suite.org/meme/tools/meme; accessed on 13 October 2025) with the parameter set to a maximum of 10 motifs to obtain the motif file. Meanwhile, the exon–intron structure information of each *NAC* gene was extracted from the genome annotation file (GFF3) using TBtools. Finally, the gene structure, protein conserved domain, and conserved motif maps were drawn using TBtools software.

For promoter cis-element analysis, the GFF file and genome file from the *T. kok-saghyz* whole-genome database were recognized using TBtools software, and the 2000 bp sequence upstream of the start codon (ATG) of each *NAC* gene was extracted as the promoter region. The promoter sequences were submitted to the PlantCARE online analysis website (https://bioinformatics.psb.ugent.be/; accessed on 26 October 2025) to predict cis-elements. The prediction results were organized, and element data without annotation, without special functions, or with unrelated functions were removed. Finally, the promoter analysis results were visualized using TBtools software to generate a distribution map of cis-elements in the promoter regions.

### 4.5. Subcellular Localization and Tissue-Specific Expression of TkNACs Proteins

Using *T. kok-saghyz* cDNA as the template (Tables S1 and S2), the CDS sequences of *TkNAC16/20/23/30* were amplified with primers containing the Sma*I* restriction site (Table S3). The fragments were inserted into the pCAMBIA1300-GFP vector by one-step cloning. After sequencing verification, the construct was transformed into *Agrobacterium tumefaciens* strain GV3101. The bacterial cells were resuspended in infiltration buffer (MgCl_2_: 2.132 g/L; MES: 2.033 g/L; sucrose: 30 g/L) to OD_600_ = 0.6 and injected into the lower epidermis of *Nicotiana benthamiana* leaves. After dark culture for 16 h, the plants were placed under normal light for 48–72 h, and co-localization of green fluorescent protein (GFP) and nuclear marker was observed using a laser scanning confocal microscope.

Roots, leaves, and flowers were collected from six-month-old *T. kok-saghyz* plants, immediately frozen in liquid nitrogen, and ground into powder. Total RNA was extracted using a polysaccharides and polyphenolics-rich kit and reverse transcribed into cDNA (Table S4). Using *T. kok-saghyz GAPDH* as an internal reference gene, the expression levels of *TkNAC16*, *TkNAC20*, *TkNAC23*, and *TkNAC30* in different tissues were analyzed by qRT-PCR.

### 4.6. Yeast Two-Hybrid (Y2H) and Bimolecular Fluorescence Complementation (BiFC)

To investigate the interactions among TkNAC proteins, Y2H and BiFC assays were performed in this study. The coding sequences of TkNAC16, TkNAC18, TkNAC20, TkNAC23, and TkNAC30 were respectively cloned into pGBKT7 and pGADT7 vectors (Table S5) via one-step cloning, and the constructs were co-transformed into the yeast Y2HGold strain. The potential toxicity of the bait vectors to the yeast strain was first examined. The empty pGBKT7 vector and pGBKT7-*TkNACs* recombinant plasmids were separately transformed into the Y2HGold strain, and the results indicate that none of the bait vectors exhibited auto-toxicity. Subsequently, the empty pGADT7 vector and each pGBKT7-*TkNACs* recombinant plasmid were co-transformed into the Y2HGold strain to test for self-activation activity. For point-to-point verification of protein interactions, the corresponding bait and prey vectors were co-transformed into the Y2HGold strain, with pGBKT7-53 + pGADT7-T as the positive control and pGBKT7-Lam + pGADT7-T as the negative control. The transformed yeast cells were plated on SD/-Trp/-Leu medium and SD/-Trp/-Leu/-His/-Ade medium, and incubated upside down at 30 °C for 3–5 days. Single colonies were picked and diluted to OD_600_ = 1.0, then spotted in 1×, 10×, 100×, and 1000× serial dilutions onto SD/-Trp/-Leu/-His/-Ade (QDO) medium and QDO medium containing X-α-gal. The plates were incubated at 30 °C for 3–5 days, and colony growth and color development were observed.

TkNAC16, TkNAC20, TkNAC23, TkNAC30, and TkNAC18 were respectively cloned into pSPYNE and pSPYCE vectors (Table S6). After sequencing verification, the constructs were transformed into *Agrobacterium tumefaciens* strain GV3101. The agrobacterial suspensions containing nYFP and cYFP fusion expression vectors were mixed in equal volumes and injected into *Nicotiana benthamiana* leaves, with co-expression of the nuclear marker protein MADS-mCherry. After dark incubation for 16 h, the plants were grown in a light incubator for 2 days (16 h light/8 h dark photoperiod). Leaf sections from the injected areas were prepared, and fluorescence signals were observed under a laser scanning confocal microscope.

### 4.7. Overexpression of TkNACs and Phenotypic Analysis of Transgenic Plants

The CDS sequences of *TkNAC16*/*20*/*23*/*30* were individually cloned into the overexpression vector pCAMBIA1300 (Table S3). After sequence verification, the constructs were transformed into *Agrobacterium rhizogenes* strain K599. The transformed strains were streaked onto TY solid plates containing kanamycin and streptomycin. Single colonies were picked for colony PCR confirmation and then activated. The bacteria were grown on the same double-antibiotic plates at 28 °C in an inverted position for 3–5 days until the bacterial lawn was confluent, and were stored for subsequent use. Hairy roots of *T. kok-saghyz* were induced using the root-cutting infiltration method. Three-month-old plants of uniform growth were selected, and a diagonal cross-cut was made 0.5 cm below the junction between the root and the leaf base, leaving only 2–3 young leaves (or young leaf buds). The plants after root cutting were co-incubated with the *Agrobacterium* infection solution containing the gene of interest in a 50 mL syringe under negative pressure to allow the bacterial solution to fully infiltrate the cut surface. Subsequently, the bacterial culture was evenly applied onto the cut surface. After PCR identification of positive plants, high-expression lines were screened by qRT-PCR. WT and transgenic plants of OE-*TkNAC16*, OE-*TkNAC20*, OE-*TkNAC23*, and OE-*TkNAC30* were transplanted into mixed potting substrate and grown in a growth chamber. Shoot and root growth were observed at 4, 8, 12, and 24 weeks, and root length was measured. Three biological replicates were set for each line. The data were analyzed using the independent sample *t*-test and one-way analysis of variance with SPSS, and graphs were plotted using GraphPad Prism 10.5 software.

### 4.8. Statistical Analysis

All experiments were conducted with at least three independent biological replicates. Quantitative data are presented as mean ± standard deviation (SD). Statistical analyses were performed using SPSS 26.0 software and GraphPad Prism 10.5 software. Comparisons between two groups (e.g., wild-type vs. overexpression lines at a single time point) were analyzed using two-tailed independent samples *t*-tests. For comparisons involving more than two groups (e.g., expression levels in different tissues or at different time points following MeJA treatment), one-way analysis of variance followed by Tukey’s post hoc test was applied. In all cases, a *p*-value < 0.05 was considered statistically significant.

## 5. Conclusions

In summary, a total of 34 NAC family members were identified from the genome of *Taraxacum kok-saghyz* (*T. kok-saghyz*), among which 27 were significantly responsive to methyl jasmonate treatment. Phylogenetic analysis, gene structure characterization, and promoter cis-element analysis indicated that the TkNAC family exhibits species-specific expansion and is extensively involved in hormone signaling and stress responses. Subcellular localization assays confirmed that TkNAC16, TkNAC20, TkNAC23, and TkNAC30 are all localized to the nucleus. Yeast two-hybrid and bimolecular fluorescence complementation assays demonstrated that TkNAC16/18/20/23/30 can form extensive heterodimers. Overexpression of *TkNAC16*, *TkNAC20*, *TkNAC23*, or *TkNAC30* significantly promoted root elongation in *T. kok-saghyz*, while leaf growth exhibited line- and stage-specific effects, indicating that these genes act as positive regulators of root architecture.

## Figures and Tables

**Table 1:** Predicted physicochemical properties and subcellular localization of the TkNAC family proteins.

Gene	Gene ID	Protein Size (aa)	Molecular Weight (Da)	pI	GRAVY	S.L.
TkNAC01	evm.model.utg1048.7	360	40,778.13	6.83	−0.512	Nucleus
TkNAC02	evm.model.utg11908.11	359	41,161.23	7.68	−0.742	Nucleus
TkNAC03	evm.model.utg12469.13	547	60,930.72	5.59	−0.604	Cytoplasm
TkNAC04	evm.model.utg12599.16	569	63,911.73	4.5	−0.592	Chloroplast
TkNAC05	evm.model.utg12599.18	400	44,847.19	5.31	−0.651	Nucleus
TkNAC06	evm.model.utg12978.15	397	44,713.64	6.41	−0.727	Vacuole
TkNAC07	evm.model.utg14059.9	340	37,633.3	8.57	−0.66	Nucleus
TkNAC08	evm.model.utg15069.2	311	35,454.98	6.54	−0.691	Nucleus
TkNAC09	evm.model.utg1654.11	351	38,830.55	7.75	−0.707	Nucleus
TkNAC11	evm.model.utg18235.9	413	47,289.57	6.41	−0.836	Nucleus
TkNAC13	evm.model.utg1925.9	461	51,810.57	4.57	−0.558	Chloroplast
TkNAC14	evm.model.utg1925.10	389	43,678.93	5.44	−0.704	Nucleus
TkNAC15	evm.model.utg24938.3	330	37,418.21	5.04	−0.638	Nucleus
TkNAC16	evm.model.utg26751.6	352	41,040.66	6.13	−0.878	Nucleus
TkNAC19	evm.model.utg3079.10	648	73,599.35	4.93	−0.72	Chloroplast
TkNAC20	evm.model.utg31242.4	286	32,476.92	8.82	−0.698	Cytoplasm
TkNAC22	evm.model.utg498.2	310	35,775.92	7.2	−0.903	Nucleus
TkNAC23	evm.model.utg522.3	441	49,685.55	6.96	−0.813	Nucleus
TkNAC24	evm.model.utg6382.4	374	41,907.58	5.53	−0.782	Peroxisome
TkNAC25	evm.model.utg6545.2	232	26,827.98	5.38	−0.809	Nucleus
TkNAC27	evm.model.utg6709.3	358	39,657.33	7.15	−0.757	Nucleus
TkNAC28	evm.model.utg8056.7	282	32,252.54	7.74	−0.716	Nucleus
TkNAC29	evm.model.utg8578.5	291	33,666.67	5.94	−0.733	Peroxisome
TkNAC30	evm.model.utg887.7	341	39,453.87	5.61	−0.791	Peroxisome
TkNAC31	evm.model.utg9118.7	420	47,418.4	6.67	−0.62	Cytoplasm
TkNAC32	evm.model.utg9212.2	238	27,479.97	7.14	−0.729	Nucleus
TkNAC33	evm.model.utg9389.11	483	53,963.41	5.49	−0.635	Nucleus

Note: TkNAC10, TkNAC12, TkNAC17, TkNAC18, TkNAC21, TkNAC26, and TkNAC34 are the other seven TkNAC family members identified in this study but they are not included in this table because they did not show significant differential expression under MeJA treatment. Protein size (aa): protein amino acid number; pI: Isoelectric point; GRAVY: Grand average of hydropathicity; S.L.: the probable subcellular location predicted by WoLF PSORT.

## Data Availability

The primers involved in this research are all in the Tables S1–S6, and the templates synthesized by the primers are all cDNA. The *T. kok-saghyz* genome and nucleotide sequences were deposited in the Genome Warehouse (GWH; http://bigd.big.ac.cn/gwh/, accessed on 18 April 2024) under the accession number PRJCA000437.

## Supplementary Materials

The following supporting information can be downloaded at: https://www.mdpi.com/article/10.3390/plants15121923/s1, Table S1: Primers used for amplifying *TkNAC16*, *TkNAC20*, *TkNAC23*, and *TkNAC30*; Table S2: Coding sequences (CDS) of *TkNAC16*, *TkNAC20*, *TkNAC23*, and *TkNAC30*; Table S3: Primers used for overexpression and subcellular localization vector construction; Table S4: Primers used for qRT-PCR analysis; Table S5: Primers used for yeast two-hybrid (Y2H) vector construction; Table S6: Primers used for bimolecular fluorescence complementation (BiFC) vector construction; and Figure S1: Leaf growth phenotypes of *TkNAC16*/*20*/*23*/*30* overexpression lines at different growth stages (related to Figure 10A).
