# Supplementary material for: TkNACs Heterodimerization and Methyl Jasmonate Signaling Synergistically Mediate Root Development in Taraxacum kok-saghyz
Source: Plants (Basel). 2026 Jun 22;15(12):1923. doi: 10.3390/plants15121923 (PMC13306433; doi:10.3390/plants15121923)
Supplement: Supplementary file 1 [file plants-15-01923-s001.zip › Supplementary figure.pdf]

Article

TkNACs Heterodimerization and Methyl Jasmonate Signaling Synergistically Mediate Root Development in *Taraxacum kok-saghyz*

Changping Zhang [1,2,†], Yixuan Lin [1,2,†], Ziting Chen [1,2,], Xiaodong Li [1,2], Yuya Geng [1,2], Jialong Sun [1,2], Lu Qiao [1,2], Xifeng Chen [1,2,*] and JieYan [1,2,*]

[1] Key Laboratory of Xinjiang Phytomedicine Resource and Utilization of Ministry of Education, College of Life Sciences, Shihezi University, Shihezi 832003, China; 15009357340@163.com (C.Z.); 17590516886@163.com (Y.L.)

[2] Xinjiang Production and Construction Corps Key Laboratory of Oasis Town and Mountain-Basin System Ecology, College of Life Sciences, Shihezi University, Shihezi 832003, China

* Correspondence: cxf_cc@shzu.edu.cn (X.C); jiey@shzu.edu.cn (J.Y.)

† These authors contributed equally to this work.

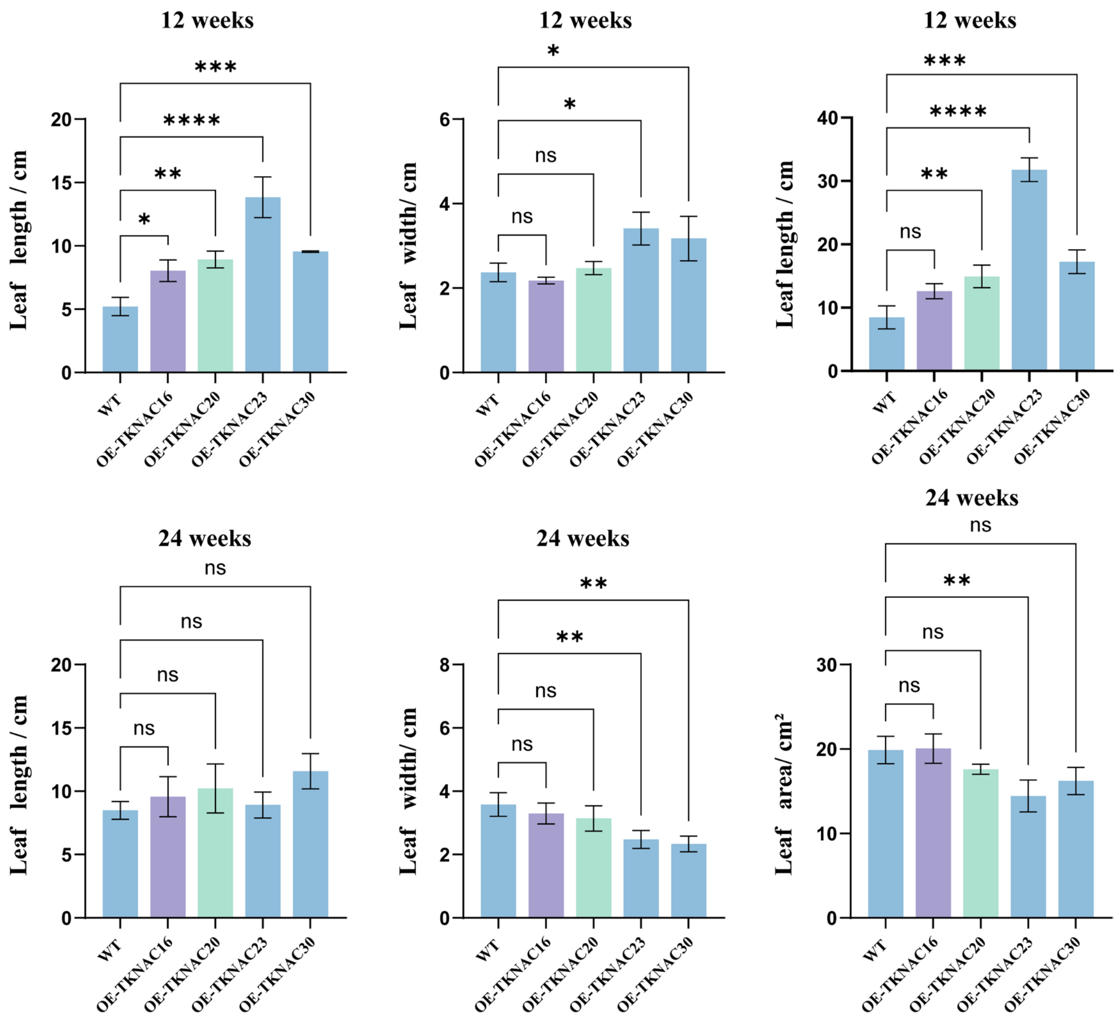

Figure S1. Leaf morphological parameters (leaf length, leaf width, and leaf area) of *Taraxacum kok-saghyz* at 12 and 24 weeks after planting. ns: no significant difference; * $p < 0.05$; ** $p < 0.01$; *** $p < 0.001$; **** $p < 0.0001$.
